# Monocyte distribution width as a promising biomarker for differential diagnosis of chronic hepatitis, cirrhosis, and hepatocellular carcinoma

**DOI:** 10.3389/fimmu.2024.1406671

**Published:** 2024-07-03

**Authors:** Sheng Lin, Xinyao Yang, Xin Yang, Minjie Tang, Xiaobao Yao, Yuchen Ye, Qunfang Huang, Jinlan Huang, Jiejuan Li, Qiang Yi, Wennan Wu, Shiqi Li, Yaru Lei, Bin Yang, Can Liu, Qishui Ou, Zhen Xun

**Affiliations:** ^1^ Department of Laboratory Medicine, Fujian Key Laboratory of Laboratory Medicine, Gene Diagnosis Research Center, Fujian Clinical Research Center for Clinical Immunology Laboratory Test, The First Affiliated Hospital, Fujian Medical University, Fuzhou, China; ^2^ Department of Laboratory Medicine, National Regional Medical Center, Binhai Campus of the First Affiliated Hospital, Fujian Medical University, Fuzhou, China; ^3^ The First Clinical College, Fujian Medical University, Fuzhou, China

**Keywords:** monocyte distribution width, chronic hepatitis B, liver cirrhosis, hepatocellular carcinoma, biomarker

## Abstract

**Objective:**

We aimed to investigate the association and diagnostic value of monocyte distribution width (MDW) for chronic hepatitis B (CHB), liver cirrhosis (LC), and hepatocellular carcinoma (HCC).

**Methods:**

MDW levels were measured in 483 individuals (103 CHB, 77 LC, 153 HCC, and 150 controls). MDW was detected using UniCel Dx900 for specific cell volume parameters and the distribution of cell volumes.

**Results:**

Our findings revealed a dynamic upward change in MDW levels across different stages of chronic liver disease, from CHB to LC and HCC. In CHB, MDW levels were highest among HBeAg-positive CHB patients and exhibited a negative correlation with HBV markers while positively correlating with ALT levels. In LC, MDW showed a positive association with the pathological progression of LC, demonstrating consistency with CP scores. MDW proved to be equally effective as traditional detection for diagnosing LC. In HCC, MDW was positively correlated with HCC occurrence and development, with higher levels observed in the high MDW group, which also exhibited elevated AFP levels, MELD scores, and 90-day mortality rates. MDW surpassed predictive models in its effectiveness for diagnosing HCC, as well as CHB and LC, with respective areas under the curve of 0.882, 0.978, and 0.973. Furthermore, MDW emerged as an independent predictor of HCC.

**Conclusion:**

MDW holds significant diagnostic efficacy in identifying CHB, LC, and HCC. These findings suggest that MDW could serve as a promising biomarker for predicting the severity of liver diseases and aid in rational clinical treatment strategies.

## 1 Introduction

The incidence, prevalence, and mortality of chronic liver disease have shown an increasing trend over the past decade (1). Chronic liver diseases result from prolonged damage to liver cells and the proliferation of fibrous tissue in the liver due to various factors including biology, chemistry, physics, and autoimmunity. These conditions encompass chronic hepatitis B (CHB), liver cirrhosis (LC), and hepatocellular carcinoma (HCC).

Currently, the diagnosis of CHB primarily relies on serological markers (2), while LC (3) and HCC (4) primarily diagnosed and monitored using imaging techniques. Despite some progress in understanding the pathological mechanisms and interventions of chronic liver diseases, effective biomarkers for predicting and preventing disease progression are still lacking. Diagnostic indicators often fail to accurately reflect the disease’s progression, and although several serum markers such as microRNAs (5, 6), duplex-linear DNA (7), phospholipase A2 (8), Sirtuin 1 (9, 10) show progressive elevation in chronic liver disease, they are not readily accessible, leading to increased costs associated with their assessment.

Monocyte distribution width (MDW) is a blood routine indicator that reflects monocyte heterogeneity, with changes in monocyte volume considered an early indicator of innate immune activation (11). Human monocytic cells in circulation can be categorized into three subsets, each showing different percentages in various diseases (12). During inflammatory conditions like sepsis (13, 14), viral infection (15, 16), and other inflammatory diseases (17, 18), the changes in the number, volume, and function of monocytes in these subsets differ. Recent research has shown that MDW has been utilized for the early detection of sepsis (19), with MDW levels increasing with the severity of the condition (11). In COVID-19 patients, MDW has been found to be highly correlated with disease deterioration and unfavorable clinical outcomes (20). However, the role of MDW in influencing liver diseases, especially HBV-related diseases, remains unknown.

In this study, we assessed MDW in CHB patients in different stages, cirrhotic patients, HCC patients, and healthy controls (HCs). We observed increasing trends in MDW across different chronic liver diseases and found a positive association between MDW levels and the natural progression of CHB, the degree of liver fibrosis, and HCC progression. These findings suggest that MDW could serve as a promising biomarker for predicting disease severity and assisting in the development of rational clinical treatments.

## 2 Materials and methods

### 2.1 Patients

**Figure 1:**
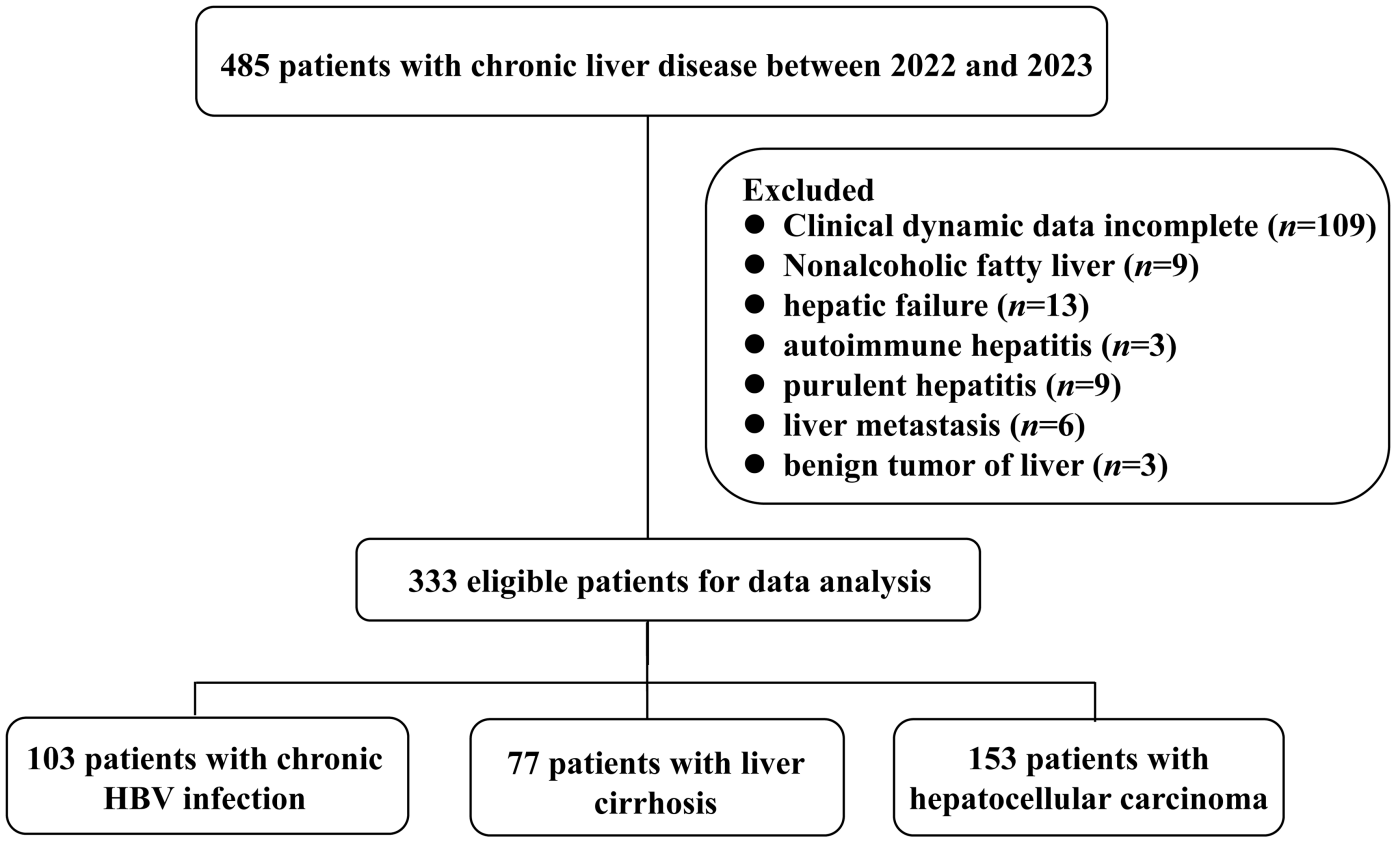
Flowchart of the patient selection process.

A total of 483 subjects were recruited from the First Affiliated Hospital of Fujian Medical University. Among them, 103 chronic HBV-infected patients were categorized into four phases based on the natural history of chronic HBV infection (21). Additionally, 77 LC patients were classified into three groups according to the Child-Pugh (CP) score: CP-A, CP-B, and CP-C (22). Furthermore, 153 HCC patients were stratified into four stages according to the China liver cancer staging (CNLC): CNLC I, CNLC II, CNLC III, and CNLC IV (4). Notably, 152 patients were excluded for various reasons (**Figure 1**). All the enrolled patients have been excluded from sepsis. Additionally, 150 healthy controls were included in this study. A summary of patient characteristics can be found in **Supplementary Tables S1**-**S4**.

All patients included in this study provided written informed consent, and the research protocol was approved by the Ethics Committee of the First Affiliated Hospital of Fujian Medical University (Approval No. MRCTA, ECFAH of FMU [2020]114).

### 2.2 Laboratory measurement of clinical indicators

The biochemical indexes were quantified by cobas® 8000 modular analyzer series (Roche diagnostics, USA). HBV DNA was detected by quantitative real-time PCR method (Sansure Biotech Inc., Hunan, China) and Roche Lightcycler 480 (Roche Corporation, Basel, Switzerland). Hepatitis B surface antigen (HBsAg), Hepatitis B e antigen (HBeAg), hepatitis B e antibody (anti-HBe), hepatitis B surface core antibody (anti-HBc), alpha-fetoprotein (AFP), and protein induced by vitamin K absence or antagonist-II (PIVKA-II) were quantified using Abbott alinity I (Abbott Laboratories, USA). International normalized ratio (INR) was detected by Sysmex CS-5100 (Sysmex, Japan). Platelet (PLT) were quantified using ADVIA2120i (siemens, Germany). MDW were quantified using UniCel Dx900 (Beckmancoulter, USA).

### 2.3 Model calculations

This study utilized multiple diagnostic models for the diagnosis of LC and HCC, which were compared with MDW. The detailed formulas are as follows:


$$\text{APRI}=\left[\text{AST }\left(\text{U}/\text{L}\right)/\text{AST }\left(\text{U}/\text{L},\text{ ULN}\right)\times 100\right]/\text{PLT }\left(\times 10^{9}/\text{L}\right)$$

(23)


$$\text{FIB}-4=\text{age }\left(\text{year}\right)\times \text{AST }\left(\text{U}/\text{L}\right)/\text{PLT }\left(\times 10^{9}/\text{L}\right)\times \text{ALT}{\left(\text{U}/\text{L}\right)}^{1/2}$$

(24)


$$\begin{array}{c} \text{MELD score}=\;3.78\times \text{ln}\left[\text{TBIL}\left(\text{μmol}/\text{L}\right)÷17.1\right]\;+\;11.2\\ \times \text{ ln }\left(\text{INR}\right)\;+\;9.57\times \text{ln }\left[\text{Cr }\left(\text{μmol}/\text{L}\right)\;÷\;88.4\right]\\ +\;6.43\left(\text{constant for liver disease etiology}\right) \end{array}$$

(25)


$$\begin{array}{c} \text{aMAP risk score }\\ =\;(\{0.06\times \text{age}+0.89\times \text{sex }\left(\text{Male}:1,\text{ Female}:0\right)+0.48\\ \times \left[\left({\text{log}}_{10}\text{TBIL }\left(\text{μmol}/\text{L}\right)\times 0.66\right)+\left(\text{albumin }\left(\text{g}/\text{L}\right)\times -0.085\right)\right]\\ -0.01\times \text{platelets}\}+7.4)/14.77\times 100 \end{array}$$

(26)


$$\begin{array}{c} \text{ASAP socre}:\text{ln}\left(\text{p}/\left(1-\text{p}\right)\right)\\ =-7.58+0.05\times \text{age}-0.58\times \text{gender }\left(\text{Male}:0,\text{ Female}:1\right)\\ +0.42\times \text{Ln }\left[\text{AFP }\left(\text{ng}/\text{ml}\right)\right]+1.11\times \text{Ln }[\text{PIVIKA}\\ -\text{II }\left(\text{mAU}/\text{ml}\right)] \end{array}$$

(27)

### 2.4 Statistical analysis

We analyzed the data utilizing GraphPad Prism 8 and SPSS 27.0.1. Statistical comparisons between two groups were conducted using the two-tailed Student’s *t*-test if quantitative variables exhibited normal and homogeneous distribution, or the Mann-Whitney *U* test if not. Differences for categorical variables were examined using the χ^2^ test. We employed Pearson correlation analysis to explore correlations, while the receiver operating characteristic (ROC) curve was utilized to compare the predictive performance of MDW between different groups. Univariate and multivariate logistic regression analyses were carried out to identify independent factors. We considered *p*-values less than 0.05 to be statistically significant.

## 3 Results

### 3.1 MDW exhibited negative correlation with serum virological markers in individuals with chronic HBV infection

**Figure 2:**
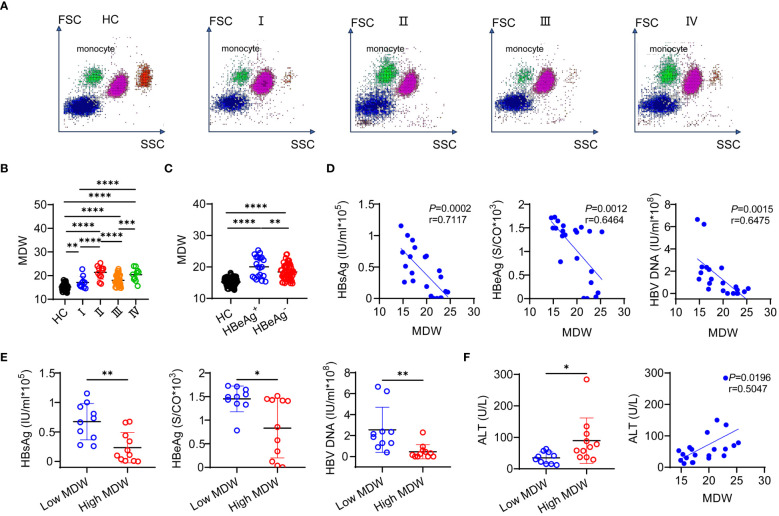
MDW differs significantly across the natural history of chronic HBV infection. **(A)** Scattergram of leukocyte throughout the HCs and four phases of chronic HBV infection. **(B)** Distribution of MDW in natural progression of chronic HBV infection and HCs (*n* = 150). I, HBeAg-positive chronic HBV infection (*n* = 10); II, HBeAg-positive CHB (*n* = 11); III, HBeAg-negative chronic HBV infection (*n* = 47); IV, HBeAg-negative CHB (*n* = 11). One-way ANOVA. **(C)** Distribution of MDW in HBeAg-positive patients (*n* = 21), HBeAg-negative patients (*n* = 58) and HCs (*n* = 150). One-way ANOVA. **(D)** Pearson correlation analysis between MDW and HBsAg, HBeAg, HBV DNA in HBeAg-positive patients (*n* = 21). **(E)** The level of HBsAg, HBeAg, HBV DNA in HBeAg-positive patients dividing into high MDW (*n* = 10) and low MDW (*n* = 11) groups. two-tailed Student’s t-test. **(F)** Pearson correlation analysis and association between MDW and ALT in HBeAg-positive patients (*n* = 21). two-tailed Student’s t-test. Data are presented as the mean ± SD. *P< 0.05, **P< 0.01, ***P< 0.001, ****P< 0.0001.

To gain insight into MDW throughout the natural history of chronic HBV infection, we analyzed MDW across different phases of the disease in patients. Our analysis revealed that monocytes exhibited a broader volume range and a more uneven longitudinal distribution in CHB patients, as depicted in the five-class scatter plot (**Figure 2A**). Notably, MDW was found to be higher in CHB patients compared to HCs, with CHB patients demonstrating higher MDW levels than those with chronic HBV infection (**Figure 2B**). Furthermore, MDW was significantly elevated in HBeAg-positive patients compared to HBeAg-negative patient, indicating that both HBeAg-positive and HBeAg-negative patients had higher MDW levels compared to HCs (**Figure 2C**). Correlation analysis revealed a strong negative correlation between MDW and three indicators in HBeAg-positive patients (**Figure 2D**). Upon dividing patients into high and low MDW groups based on equal patient numbers, we observed significantly elevated levels of HBsAg, HBeAg, and HBV DNA in the low MDW group compared to the high MDW group in HBeAg-positive patients (**Figure 2E**). Additionally, a significant correlation was noted ALT levels and MDW, with higher ALT levels observed in the high MDW group compared to the low MDW group (**Figure 2F**). While there was no significant correlation between MDW and HBsAg, HBeAg, HBV DNA, ALT levels in immune-inactive phase patients (**Supplementary Figure S1A**) and immune-active phase patients (**Supplementary Figure S1B**).

Taken together, these findings suggest differential distribution of MDW across different phases of chronic HBV infection. MDW exhibits a negative correlation with serum HBV virological markers and a positive correlation with ALT levels. Thus, incorporating measurements of both ALT and MDW could be valuable in assessing liver inflammation, particularly in HBeAg-positive individuals.

### 3.2 MDW showed positive association with pathological progression of LC

LC, which represents the late stage of progressive hepatic disease, poses a significant threat to global mortality rates. Identifying high-risk patients necessitates the utilization of prognostic indicators. The CP score has gained widespread acceptance for evaluating the severity of liver dysfunction in clinical settings. Here, we analyzed the relationship between MDW and CP classification to assess the efficacy of MDW in predicting LC progression.

**Figure 3:**
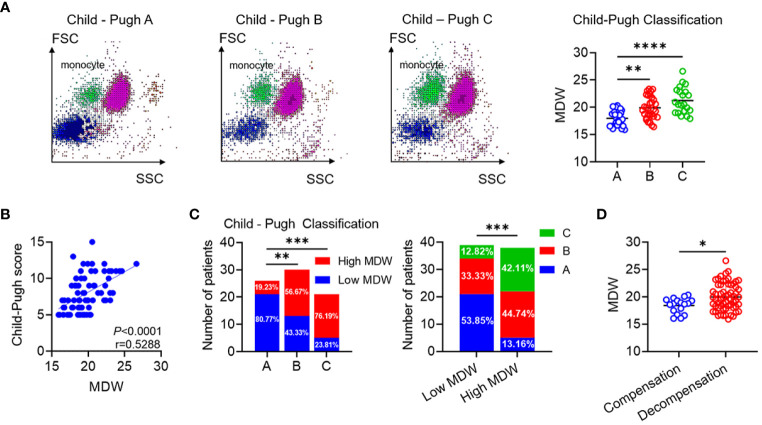
MDW is positively associated with pathological progression of liver cirrhosis. **(A)** Typical scattergram of leukocyte throughout the A, B and C Group. Comparison of MDW levels according to the CP classification (A: *n*=26, B: *n*=30, C: *n*=21). One-way ANOVA. **(B)** Analysis of the correlation between MDW and CP score in patients with cirrhosis (*n* = 77). **(C)** Number of low MDW and high MDW patients in the CP scores of A (*n* = 26), B (*n* = 30) and C (*n* =21) group. The number of CP score A, B and C patients in low MDW (*n* = 39) and high MDW (*n* = 38) groups. Chi-squared test. **(D)** Comparison of MDW levels according to the phase of compensation (*n*=17) and decompensation (*n* =60). two-tailed Student’s t-test. Data are means ± SD. *P< 0.05, **P< 0.01, ***P< 0.001, ****P< 0.0001.

In the scattergram depicting leukocyte distribution across the three stages, noticeable elevation was evident when comparing groups B and C with group A. Furthermore, the more scattered points representing monocytes suggested increased volumetric heterogeneity (**Figure 3A**). Analyzing the correlation between MDW and CP score in LC patients revealed a positive correlation (**Figure 3B**). Subsequently, we compared the composition ratios of high and low MDW across the three stages to delve deeper into the relationship between MDW and CP classification. The results demonstrated a progressive increase in the ratio of high MDW levels across stages A, B, and C. Notably, stage A exhibited a relatively higher proportion of low MDW levels, while the ratio of stage C was significantly elevated among those with high MDW levels (**Figure 3C**). Additionally, MDW levels were notably higher in the decompensated phase compared to the compensated phase (**Figure 3D**). However, there was no significant difference in MDW levels between HBV- related LC patients and non-HBV-related LC patients (**Supplementary Figure S2A**).

Collectively, our findings suggest a parallel increase in MDW with the pathological progression of LC. MDW shows promise as a novel non-invasive indicator for detecting LC progression.

### 3.3 MDW demonstrated positive correlation with both severity and grade of HCC

**Figure 4:**
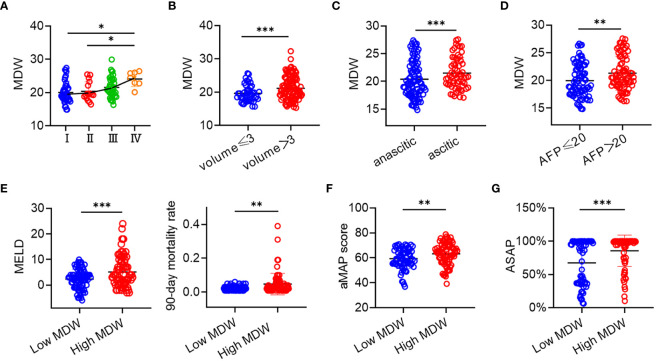
MDW was related to the severity and grade of HCC. **(A)** Distribution of MDW in CNLC I, II, III, and IV (I: *n*=40, II: *n*=16, III: *n*=39, IV: *n*=7). **(B)** MDW correlates with the size of liver tumor including volumes<3 (*n*=45) and volumes>3 (*n*=108). **(C)** MDW correlates with both ascitic (*n*=91) and anascitic HCC (*n*=62). **(D)** MDW associates with AFP of less than 20 (*n*=75) and AFP of greater than 20 (*n*=78). **(E)** MDW associates with MELD and 90-day mortality rate in low MDW (*n*=73) and high MDW (*n*=80) groups. **(F)** MDW associates with aMAP in low MDW (*n*=73) and high MDW (*n*=80) groups. **(G)** MDW associates with ASAP in low MDW (*n*=61) and high MDW (*n*=73) groups. Data are means ± SD. two-tailed Student’s t-test. *P< 0.05, **P< 0.01, ***P< 0.001.

Macrophages in the liver partially originate from monocytes circulating in the blood. Studies have indicated that macrophages undergo polarization with in the tumor microenvironment (28). It is widely accepted that M2 macrophages promote tumor progression, whereas M1 macrophages display anti-tumor activities, indicating notable functional and metabolic disparities (29). To systematically characterize the stages of HCC, we adopted the CNLC, dividing HCC into four stages: I, II, III, and IV. Higher stages correspond to increased severity of HCC. Monocyte scattergrams exhibited an uneven distribution across stages, with a corresponding rise in MDW. Moreover, MDW showed a progressive increase across the four distinct stages (**Figure 4A**). To ascertain the correlation between MDW and HCC severity, we enrolled 31 patients with liver tissue pathological sections. Our observations revealed a positive correlation between MDW and HCC severity, evidenced by tumor size and ascites formation (**Figures 4B, C**). AFP is typically produced at low levels in healthy individuals (30) and plays a crucial role in liver diseases, particularly in primary HCC. To delve deeper into the impact of MDW on HCC patients, we stratified patients into two groups based on AFP reference range. We found that MDW levels were higher in the high AFP group compared to the low AFP group, as depicted in **Figure 4D**. However, there was no significant difference in MDW levels between HBV-related HCC patients and non-HBV-related HCC patients (**Supplementary Figure S2B**).

Furthermore, to assess the predictive value of MDW for HCC, we correlated MDW with HCC-related prediction models. The MELD score, incorporating INR, creatinine, and bilirubin, was examined among HCC patients. Those in the high MDW group exhibited elevated MELD scores and a higher 90-day mortality rate compared to those in the low MDW group, as shown in **Figure 4E**. The aMAP score is a reliable risk predictor for HCC development, irrespective of etiology and ethnicity. Combining the aMAP risk score with MDW revealed a higher aMAP score in the high MDW group (**Figure 4F**). Additionally, the ASAP model is utilized to evaluate the prevalence risk in HBV-associated HCC patients. We observed a higher ASAP score in the high MDW group compared to the low MDW group (**Figure 4G**).

These findings underscore that MDW increases with the progression of HCC, and the predictive value of HCC-related models is significantly enhanced in patients with high MDW levels compared to those with low MDW levels.

### 3.4 Predictive value of MDW for different stages in patients with CHB, LC, and HCC

We utilized logistic regression analysis to identify predictors of MDW. A univariate analysis model constructed in this study verified three protective effects of Gender, Total protein, and Albumin to predict HCC (**Table 1**). Conversely, age, AFP, PIVKA-II, aMAP, ASAP, Total bilirubin, ALT, AST and MDW (OR=4.64, *P*<0.001) were suggested as detrimental to HCC prediction (**Table 1**). Multivariate logistic regression analysis, excluding eight collinearity variables among the significant variables, identified twelve parameters influencing HCC. AFP, aMAP, and MDW (OR=3.57, *P*<0.001) were still suggested as detrimental to predict HCC (**Table 1**).

**Table 1:** Logistic regression analysis to predict MDW in HCC.

Baseline variables	Univariate analysis		Multivariate analysis	
	OR (95% CI)	P value	OR (95% CI)	*P* value
**Gender**	0.26(0.15–0.45)	<0.001	0.42(0.11–1.64)	0.208
**age**	1.70(1.39–2.06)	<0.001	–	–
**AFP, ng/ml**	1.90 (1.53–2.37)	<0.001	1.66 (1.01–2.72)	0.044
**PIVKA-II, mAU/ml**	1.10 (1.06–1.15)	<0.001	–	–
**aMAP**	1.23 (1.17–1.28)	<0.001	1.20(1.09–1.32)	<0.001
**ASAP**	20.04(7.39–54.37)	<0.001	–	–
**Total bilirubin, μmol/L**	1.11(1.07–1.16)	<0.001	–	–
**Total protein, g/L**	0.75(0.70–0.80)	<0.001	–	–
**Albumin, g/L**	0.44(0.35–0.55)	<0.001	–	–
**ALT, U/L**	1.08(1.05–1.10)	<0.001	–	–
**AST, U/L**	1.32(1.23–1.42)	<0.001	–	–
**MDW**	4.64 (3.14–6.83)	<0.001	3.57 (2.11–6.03)	<0.001

Bold font indicates statistical significance (P< 0.05).

Odds ratio (OR) > 1 indicates a detrimental association with HCC. OR< 1 indicates a protective association with HCC. AFP, alpha-fetoprotein; ALT, alanine aminotransferase; AST, aspartate aminotransferase; MDW, monocyte distribution width.

**Figure 5:**
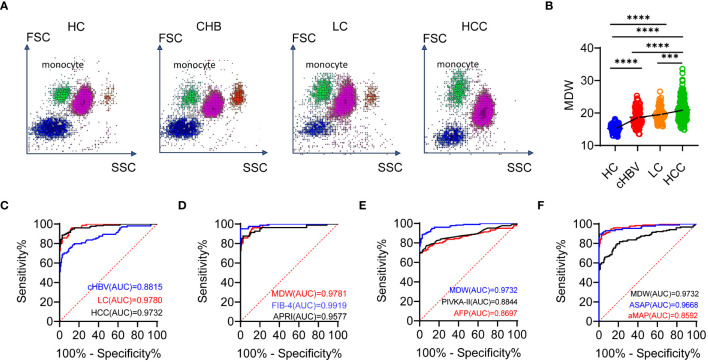
MDW differs significantly across the chronic HBV infection, LC and HCC. **(A)** Scattergram of leukocyte throughout the chronic HBV infection (cHBV, *n* = 103), LC (*n* = 77), HCC (*n* = 153) and HCs (*n* = 150). **(B)** Distribution of MDW throughout chronic HBV infection, LC, HCC and HCs. One-way ANOVA. **(C)** ROC curves of MDW in HCs, CHB, LC and HCC. **(D)** ROC curves of MDW, APRI and FIB-4 in predicting liver cirrhosis. **(E)** ROC curves of MDW, AFP and PIVKA-II in predicting HCC. **(F)** ROC curves of MDW, ASAP and aMAP in predicting HCC. ***P< 0.001, ****P< 0.0001.

The scattergram of leukocyte throughout chronic HBV infection, LC, HCC, and HCs depicted the distribution of cells in different diseases. We observed that the distribution of monocytes became increasingly uneven as the disease progressed, indicating marked heterogeneity in volumetric analysis (**Figure 5A**). Additionally, the results demonstrated the dynamic upward change of MDW in different stages of chronic liver disease, ranging from CHB, LC to HCC (**Figure 5B**).

To further evaluate the value of MDW in comparison to predictive power, we performed receiver operating characteristic (ROC) curve analysis. For CHB, LC and HCC, the area under the curve (AUC) (0.882, 95% CI: 0.836–0.927; 0.978, 95% CI: 0.964–0.993; 0.973, 95% CI: 0.958–0.988) indicated significant specificity and sensitivity in predicting MDW (**Figure 5C**). The AUC of MDW showed comparable prediction with the fibrosis-4 index (FIB-4) (0.992, 95% CI: 0.983–1.000) in LC but was higher than that of AST to platelet ratio index (APRI) (0.958, 95% CI: 0.925–0.990) (**Figure 5D**). Regarding the prediction indicators of HCC, the AUC of MDW was higher than that of AFP (0.866, 95% CI: 0.822–0.910), PIVKA-II (0.884, 95% CI: 0.840–0.929) as well as two HCC prediction models aMAP (0.859, 95% CI: 0.816–0.902) and ASAP (0.967, 95% CI: 0.939–0.994) (**Figures 5E, F**).

These results suggested that MDW significantly differs in CHB, LC and HCC. The predictive capability of MDW in LC is comparable to APRI and FIB-4, but superior to AFP and even aMAP, MELD, and ASAP models in predicting HCC.

## 4 Discussion

Our study highlights the significance of MDW in predicting chronic liver diseases. We have found that MDW serves as a reliable indicator of liver inflammation severity. Specifically, we observed a significant increase in MDW values in patients with CHB, potentially linked to the activation of monocytes induced by liver inflammation. The appearance of inflammation coincides with a decrease in HBsAg, HBeAg, and HBV DNA. Host immunity clears HBV-infected hepatocytes when viral infection occurs, leading to the elevation of ALT levels. Simultaneously, the clearance of HBV-infected hepatocytes results in decreased HBsAg, HBeAg, and HBV DNA levels (31). Notably, as liver inflammation progresses towards irreversible damage or cirrhosis, MDW levels continue to rise steadily. In the progression to HCC, several indicators and diagnostic models suggest a notable association between MDW and the severity of HCC. These findings reveal that monocytes undergo continuous changes throughout tumor progression, resulting in alterations in volume and functions. As the anti-tumor activity of monocytes diminishes with the advancement of HCC, monocytes with different phenotypes are recruited to the injured liver via chemokine signals (32), thereby impacting the MDW. Hence, based on MDW levels, we offer a means of assessing various stages of disease progression in CHB, LC, HCC, and related conditions.

MDW reflects the variation in monocyte volume, with higher values indicating greater differences in monocyte size. The volume, conductivity, light scatter technique not only measures the size and internal structure of monocytes but also allows for the evaluation of cytoplasmic granularity and nuclear structure. The resulting MDW value provides a quantitative evaluation of morphological changes in activated monocytes, aiding in assessing the current innate immune capacity and analyzing monocyte polarization trends (33). Several potential mechanisms underlie the use of MDW as a diagnostic marker for liver disease. Firstly, liver disease often accompanies inflammation and cell damage, leading to monocyte activation and changes in monocyte size distribution. Secondly, MDW can be measured through routine complete blood count testing, offering the advantages of convenience and affordability. Although the relationship between MDW and liver diseases has not been fully investigated, prior studies have shown associations between MDW and chronic inflammation, as well as its sensitivity and specificity in response to infection (11).

Currently, the clinical diagnosis and staging of HBV infection involve a comprehensive evaluation of markers including HBsAg, HBeAg, HBV DNA, and ALT levels. Although ALT is commonly used to assess liver inflammation, its correlation with liver inflammation is not entirely consistent, as individuals with normal ALT levels can still exhibit severe liver fibrosis. Hence, there is a growing demand for novel inflammatory markers in clinical practice. However, it has been observed that MDW levels show a noticeable elevation during liver inflammation, and the extent of this increase can provide insights into the severity of the inflammatory response within the liver. Therefore, MDW can be considered a valuable diagnostic tool for staging CHB.

Liver biopsy is regarded as the gold standard for diagnosing cirrhosis. However, it is increasingly being replaced by noninvasive methods such as serologic measures and imaging-based indices. The FIB-4 (24) and APRI (23), developed based on chronic HCV-infected patients, are widely accepted tools for indirect signs of liver fibrosis and dysfunction. However, their application to other types of cirrhosis is often nonspecific or insensitive (34). The AUC of MDW showed comparable predictive ability with FIB-4 and APRI. Furthermore, the progression of fibrosis renders the liver sinusoids specialized macrophages, Kupffer cells, incapable of clearing bacteria, which can stimulate the recruitment of monocytes to form large aggregates of multinucleated cells that display enhanced bacterial capture ability (35). This may result in elevated MDW levels in LC. The risk of cirrhosis can be assessed by observing elevated MDW on routine blood tests. In addition, the ratio of monocyte to high-density lipoprotein cholesterol was positively associated with the risk of significant liver fibrosis and cirrhosis (36). Therefore, the diagnostic value of MDW combined with other biomarkers on the severity of liver diseases warrants further study.

The diagnosis of HCC currently relies on imaging techniques, pathology, and molecular diagnostic technology. Laboratory indicator testing offers superior diagnostic capabilities for disease progression while minimizing physical harm to patients compared to other methods. However, the laboratory diagnosis of HCC mainly depends on AFP, which is not consistently elevated in some HCC patients. It is noteworthy that some studies have suggested a higher false positive rate and suboptimal diagnostic efficacy associated with AFP diagnosis (37).

Although numerous HCC prediction models exist, they primarily focus on various cytokines secreted by different types of cells, which may lag behind cellular changes. Therefore, MDW can provide a valuable reference for the diagnosis of HCC. Macrophages are the predominant stromal cells found in the microenvironment of HCC. Studies have shown that the secretion of pyruvate kinase M2 by HCC cells leads to monocyte differentiation into macrophages and remodeling of the tumor microenvironment. This ultimately enhances the invasiveness of HCC (38). These macrophages primarily originate from circulating monocytes and undergo maturation upon stimulation by regulators secreted by tumor cells (39). Therefore, according to past data and literature, we infer that monocytes exhibit morphological and functional changes in the early stages of HCC, which can be intuitively reflected by MDW.

Certain limitations of our study should be mentioned. First, the mechanisms involved in how MDW impact chronic liver disease deserve further study. Second, more research is needed to prove whether is meaningful in other types of chronic liver diseases in subsequent studies, such as autoimmune hepatitis, nonalcoholic fatty liver disease, HBV-related acute-on-chronic liver failure, and so on. Third, the number of patients with HCC needs to be further enlarged.

In conclusion, MDW serves as a valuable and novel biomarker, allowing for the assessment of HBV infection and liver inflammatory damage in CHB, degree of liver fibrosis, and prediction of HCC progression. The application of MDW can assist clinicians in managing chronic HBV infection, LC, and HCC by assessing disease severity and developing appropriate clinical treatments.

## Data availability statement

The original contributions presented in the study are included in the article/**Supplementary Material**. Further inquiries can be directed to the corresponding authors.

## Ethics statement

The studies involving humans were approved by the Ethics Committee of the First Affiliated Hospital of Fujian Medical University. The studies were conducted in accordance with the local legislation and institutional requirements. The participants provided their written informed consent to participate in this study.

## Author contributions

SL: Data curation, Writing – original draft. XYY: Formal analysis, Data curation, Writing – original draft. XY: Formal analysis, Data curation, Writing – original draft. MJT: Writing – review & editing, Data curation. XBY: Writing – review & editing, Data curation. YCY: Writing – review & editing. QFH: Writing – review & editing, Methodology. JLH: Writing – review & editing, Formal analysis. JJL: Writing – review & editing, Data curation. QY: Writing – review & editing, Formal analysis. WNW: Writing – review & editing, Data curation. SQL: Writing – review & editing, Methodology. YRL: Writing – review & editing, Data curation. BY: Writing – review & editing, Supervision. CL: Writing – review & editing, Supervision. QSO: Funding acquisition, Writing – review & editing. ZX: Formal analysis, Data curation, Writing – original draft, Funding acquisition, Writing – review & editing.
