# Supplementary material for: Monocyte distribution width as a promising biomarker for differential diagnosis of chronic hepatitis, cirrhosis, and hepatocellular carcinoma
Source: Front Immunol. 2024 Jul 3;15:1406671. doi: 10.3389/fimmu.2024.1406671 (PMC11251903; doi:10.3389/fimmu.2024.1406671)
Supplement: Supplementary file 1 [file DataSheet_1.docx]

**Monocyte distribution width as a promising biomarker for differential diagnosis of chronic hepatitis, cirrhosis, and hepatocellular carcinoma**

Sheng Lin^1,2,3#^, Xinyao Yang^1,3#^, Xin Yang^1,3#^, Minjie Tang^1,2^, Xiaobao Yao^1,3^, Yuchen Ye^1,3^, Qunfang Huang^1,2^, Jinlan Huang^1,2,3^, Jiejuan Li^1,2^, Qiang Yi^1,2^, Wennan Wu^1,2,3^, Shiqi Li^1,2^, Yaru Lei^3^, Bin Yang^1,2,3^, Can Liu^1,2,3^, Qishui Ou^1,2,3*^, Zhen Xun^1,2,3*^

^1^ Department of Laboratory Medicine, Fujian Key Laboratory of Laboratory Medicine, Gene Diagnosis Research Center, Fujian Clinical Research Center for Clinical Immunology Laboratory Test, The First Affiliated Hospital, Fujian Medical University, Fuzhou, China

^2^ Department of Laboratory Medicine, National Regional Medical Center, Binhai Campus of the First Affiliated Hospital, Fujian Medical University, Fuzhou, China

^3^ The First Clinical College, Fujian Medical University, Fuzhou, China.

^#^ These authors contributed equally to this work.

* **Corresponding author:** Qishui Ou, MD, PhD, Department of Laboratory Medicine, The First Affiliated Hospital, Fujian Medical University, 20 Chazhong Road, Fuzhou 350005, Fujian, China. E-mail: [ouqishui@fjmu.edu.cn](mailto:ouqishui@fjmu.edu.cn). or Zhen Xun, MD, PhD, Department of Laboratory Medicine, The First Affiliated Hospital, Fujian Medical University, 20 Chazhong Road, Fuzhou 350005, Fujian, China. E-mail: [xunzhen@fjmu.edu.cn](mailto:xunzhen@fjmu.edu.cn). Tel: +86-591-87981969; Fax: 86-591-83340702.

**Tables of content**

Table S1 .........................................................................................................................3

Table S2..........................................................................................................................5

Table S3..........................................................................................................................6

Table S4..........................................................................................................................7

Figure S1........................................................................................................................8

Figure S2........................................................................................................................9

**Table S1. Baseline demographics and clinical characteristics of the study population.**

| Variables | HC | CHB | LC | HCC |
| --- | --- | --- | --- | --- |
| Number of participants | 150 | 103 | 77 | 153 |
| Clinical parameters |  |  |  |  |
| Gender, *n* Male/*n* Female | 80/70 | 67/36 | 64/17 | 130/23 |
| Age, years | 32±7 | 43±12 | 55±11 | 59±12 |
| Laboratory parameters |  |  |  |  |
| Log_10_HBsAg, IU/ml | – | 3.14±1.23 | – | – |
| Log_10_HBeAg, S/CO | – | 0.56±1.45 | – | – |
| HBeAb, S/CO | – | 9.66±18.79 | – | – |
| Log_10_HBV DNA, IU/ml | – | 4.06±2.42 | – | – |
| ALT, U/L | 18±9.02 | 60±168.96 | – | – |
| APRI | – | – | 2.90±8.17 | – |
| FIB-4 | – | – | 7.89±10.59 | – |
| AFP | 2.19±0.92 | – | – | 4234.88±13559.32 |
| PIVKA | 20.09±5.84 | – | – | 7037.38±16923.49 |
| MELD | – | – | – | 3.78±5.26 |
| 90 days mortality rate (%) | – | – | – | 3.6±4.8 |
| aMAP | 50.37±5.22 | – | – | 61.33±8.59 |
| ASAP | 8.55±4.61 | – | – | 77.74±29.66 |
| MDW | 15.22±1.18 | 18.78±2.96 | 19.66±2.40 | 20.69±3.28 |

Data are mean ± SD unless otherwise indicated. HBV, hepatitis B virus; HBeAg, hepatitis B e antigen; HBsAg, hepatitis B surface antigen; ALT, alanine aminotransferase; APRI, Aspartate aminotransferase-to-Platelet Ratio Index; FIB-4, Fibrosis 4 score AFP, alpha-fetoprotein; PIVKA-Ⅱ, vitamin K absence or antagonist-II; MELD, Model for end-stage liver disease; ASAP, model based on age, sex, AFP, and PIVKA-II; aMAP, age–male–ALBI–platelets; MDW, Monocyte distribution width.

**Table S2. Baseline demographics and clinical characteristics of the study population in CHB.**

| Variables | **HBeAg-positive** **chronic HBV infection** | **HBeAg-positive chronic**  **hepatitis B** | **HBeAg-negative chronic HBV infection** | **HBeAg-negative chronic hepatitis B** |
| --- | --- | --- | --- | --- |
| Number of participants | 10 | 11 | 47 | 11 |
| **Clinical parameters** |  |  |  |  |
| Gender, *n* Male/*n* Female | 9/1 | 5/6 | 29/18 | 9/2 |
| Age, years | 38±10 | 34±13 | 47±11 | 43±12 |
| **Laboratory parameters** |  |  |  |  |
| Log_10_HBsAg, IU/ml | 4.78±0.21 | 3.66±1.13 | 2.82±0.94 | 2.60±1.19 |
| Log_10_HBeAg, S/CO | 3.18±0.04 | 2.54±0.79 | -0.38±0.23 | -0.31±0.36 |
| HBeAb, S/CO | 56.62±2.26 | 26.47±15.16 | 0.24±0.47 | 0.06±0.12 |
| Log_10_HBV DNA, IU/ml | 8.25±0.33 | 5.99±2.22 | 2.60±1.07 | 4.61±0.98 |
| ALT, U/L | 29±12.69 | 88±6.27 | 20±8.89 | 143±240.39 |
| MDW | 17.07±2.41 | 21.60±2.72 | 17.97±2.10 | 18.53±2.57 |

Data are mean ± SD unless otherwise indicated. HBV, hepatitis B virus; HBeAg, hepatitis B e antigen; HBsAg, hepatitis B surface antigen; ALT, alanine aminotransferase; MDW, monocyte distribution width.

**Table S3. Baseline demographics and clinical characteristics of the study population in LC.**

| Variables | **CP-A** | **CP-B** | **CP-C** |
| --- | --- | --- | --- |
| Number of participants | 26 | 30 | 21 |
| **Clinical parameters** |  |  |  |
| Gender, *n* Male/*n*, Female | 21/5 | 25/5 | 18/3 |
| Age, years | 57±12 | 54±12 | 51±11 |
| **Laboratory parameters** |  |  |  |
| APRI | 0.71±7.21 | 3.78±7.73 | 3.41±7.58 |
| FIB-4 | 3.52±9.99 | 9.97±10.50 | 11.45±10.39 |
| MDW | 17.96±2.34 | 19.92±2.38 | 21.25±2.36 |

Data are mean ± SD unless otherwise indicated. CP, Child-Pugh score; APRI, Aspartate aminotransferase-to-Platelet Ratio Index; FIB-4, Fibrosis 4 score; MDW, monocyte distribution width.

**Table S4. Baseline demographics and clinical characteristics of the study population in HCC.**

| Variables | **CNLN Ⅰ** | **CNLN Ⅱ** | **CNLN Ⅲ** | **CNLN Ⅳ** |
| --- | --- | --- | --- | --- |
| Number of participants | 40 | 16 | 40 | 7 |
| **Clinical parameters** |  |  |  |  |
| Gender, *n* Male/*n* Female | 34/6 | 16/0 | 35/5 | 6/1 |
| Age, years | 61±11 | 61±11 | 58±11 | 59±12 |
| **Laboratory parameters** |  |  |  |  |
| AFP | 4346±18198 | 4594±16604 | 13041±18044 | 3416±17546 |
| PIVKA | 3994.04±20372.92 | 7354.14±20506.35 | 15900.91±20783.03 | 7027±14886.72 |
| MELD | 5.51±5.41 | 4.66±5.26 | 4.88±5.47 | 14.82±5.98 |
| aMAP | 61.19±9.15 | 62.48±9.05 | 61.59±9.13 | 64.75±9.50 |
| MDW | 20.32±3.38 | 20.33±3.27 | 21.58±3.15 | 24.04±3.26 |

Data are mean ± SD unless otherwise indicated. CNLC, China HCC staging; AFP, alpha-fetoprotein; PIVKA-Ⅱ, vitamin K absence or antagonist-II; MELD, Model for end-stage liver disease; aMAP, age–male–ALBI–platelets; MDW, monocyte distribution width.


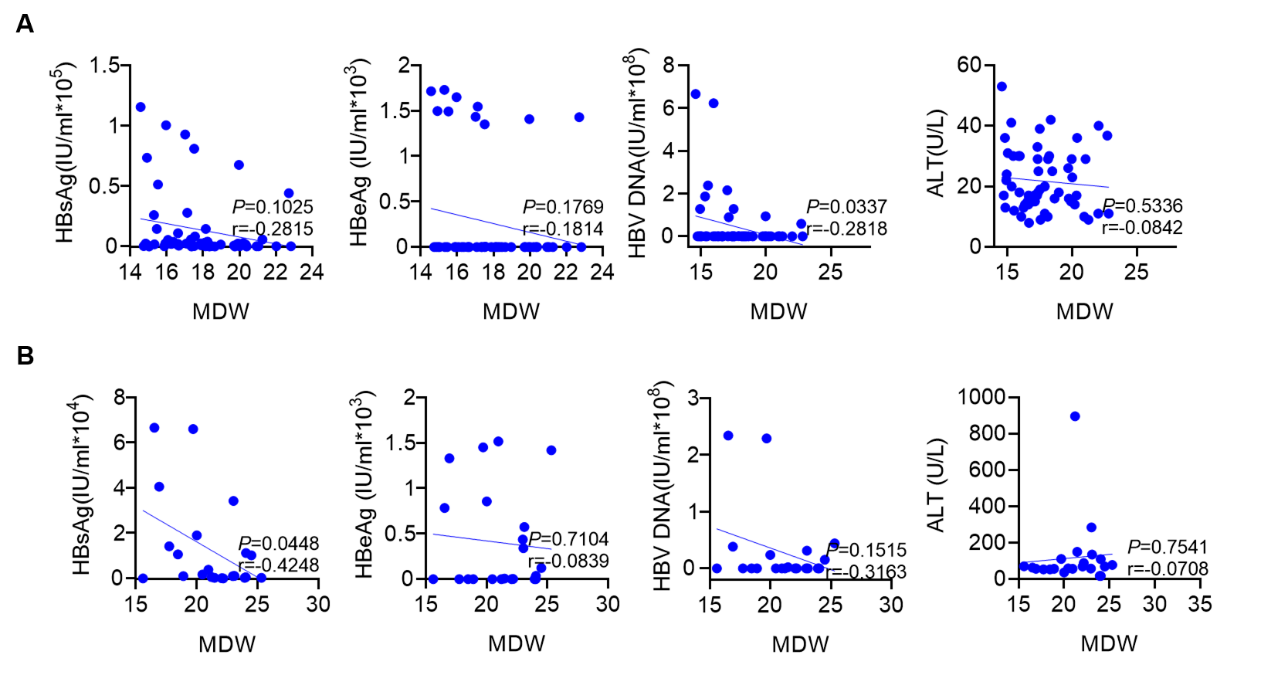


**Figure S1.** **Correlation between MDW and HBsAg, HBeAg, HBV DNA, ALT levels in** **immune-inactive phase and immune-active phase** **patients.** **(A)** Pearson correlation analysis between MDW and HBsAg, HBeAg, HBV DNA, ALT levels in immune-inactive phase patients (*n* = 57). **(B)** Pearson correlation analysis between MDW and HBsAg, HBeAg, HBV DNA, ALT levels in immune-active phase patients (*n* = 22).


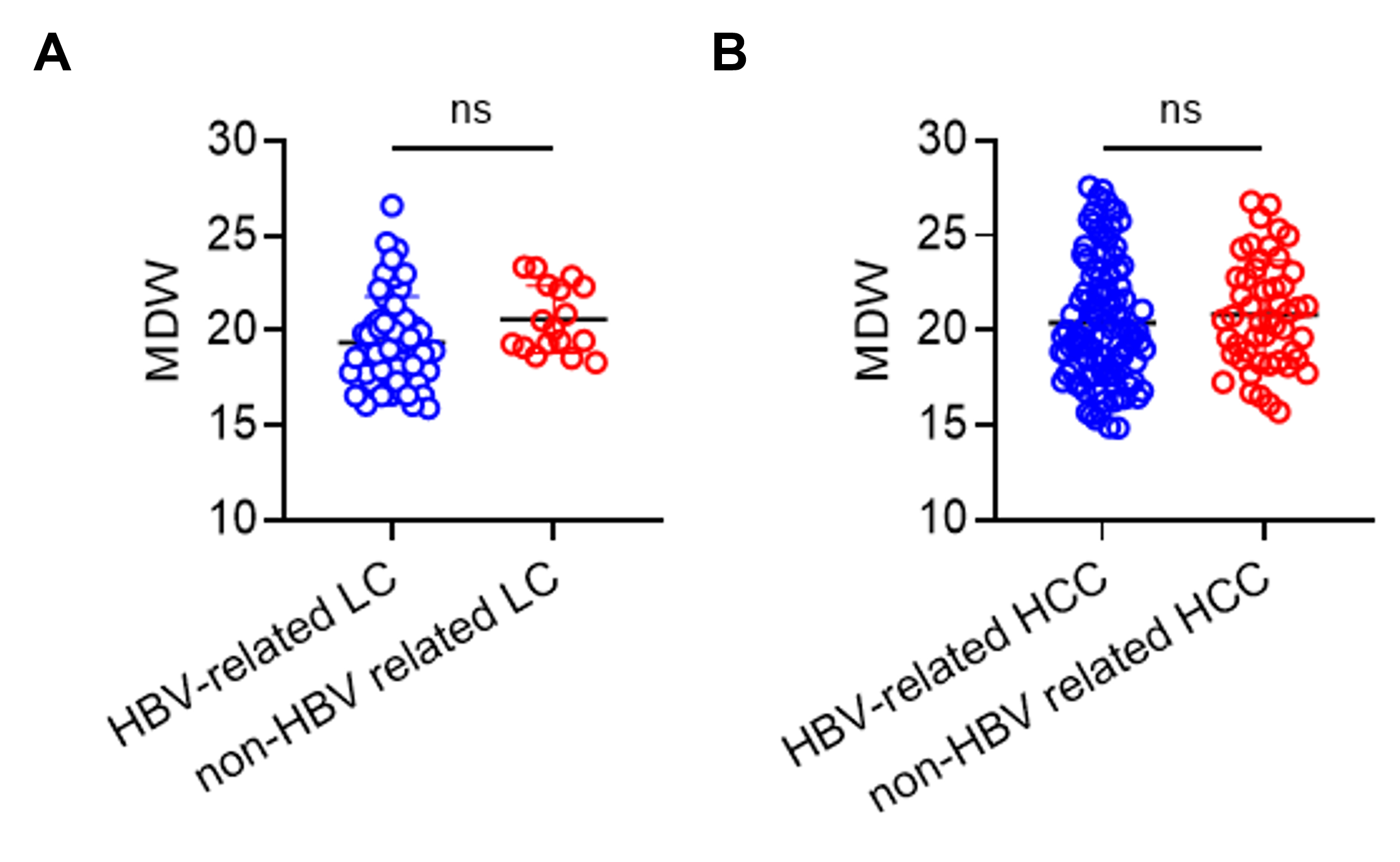


**Figure S2. The MDW level between HBV-related and non-HBV-related LC and HCC patients. (A)** MDW in patients with HBV-related LC (*n*=60) and non-HBV-related LC (*n*=17). **(B)** MDW in patients with HBV-related HCC (*n*=103) and non-HBV-related HCC (*n*=50).
